# Myocardial perfusion MRI using SW-CG-HYPR for the detection of coronary artery disease

**DOI:** 10.1186/1532-429X-14-S1-T5

**Published:** 2012-02-01

**Authors:** Jun Yang, Heng Ma, Jing Liu, Lan Ge, David Chen, Kai Lin, Jing An, Lixin Jin, Kuncheng Li, Debiao Li

**Affiliations:** 1 Yuhuangding Hospital, Yantai, China; 2 Capital Medical University, Beijing, China; 3 University of California, Los Angeles, CA, USA; 4 Siemens Mindit Magnetic Resonance, Shenzhen, China; 5 Siemens Limited China, Shanghai, China

## Summary

In this work, we have prospectively examined the diagnostic value of adenosine-induced stress myocardial perfusion MRI using SW-CG-HYPR in 50 patients with suspected CAD. Using the high-resolution, whole left ventricle SW-CG-HYPR method, perfusion MRI was able to depict hemodynamically relevant coronary artery stenosis with an accuracy of 90% and 93% based on per-patient and per-vessel analyses, respectively, using x-ray coronary angiography as the reference standard.

## Background

Myocardial perfusion MRI with SW-CG-HYPR allows increased spatial coverage (whole left ventricular coverage), improved temporal and spatial resolution and signal-to-noise ratio, and reduced motion artifacts. The accuracy of this technique for detecting coronary artery disease (CAD) has not been determined in a large number of patients. The purpose of this study was to prospectively evaluate the diagnostic performance of myocardial perfusion MRI with SW-CG-HYPR in patients with suspected CAD.

## Methods

Fifty consecutive patients (28 men and 22 women; mean age, 56 ± 16 years) who were scheduled for coronary angiography with suspected CAD underwent myocardial perfusion MRI with SW-CG-HYPR at 3.0T. Perfusion defects were interpreted qualitatively by 2 blinded observers and were correlated to x-ray angiographic stenoses ≥ 50%.

## Results

The prevalence of CAD was 56%. In the per-patient analysis, the sensitivity, specificity, positive predictive value, negative predictive value, and accuracy of SW-CG-HYPR myocardial perfusion imaging were 96% (95% confidence interval [CI] 82% to 100%), 82% (95% CI 60% to 95%), 87% (95% CI 70% to 96), 95% (95% CI 74% to100%), and 90% (95% CI 82% to 98%), respectively. In the per-vessel analysis, these values were 98% (95% CI 91% to 100%), 89% (95% CI 80% to 94%), 86% (95% CI 76% to 93%), 99% (95% CI 93% to 100%), and 93% (95% CI 89% to 97%), respectively.

## Conclusions

Myocardial perfusion MRI using SW-CG-HYPR allows whole left ventricular coverage and high resolution, and has high diagnostic accuracy in patients with suspected CAD.

## Funding

This work was supported by National Institute of Health (NIBIB EB002623) and National Natural Science Foundation of China (30828009).

